# Celiac disease and bone

**DOI:** 10.20945/2359-3997000000561

**Published:** 2022-11-10

**Authors:** Ananya V. Kondapalli, Marcella Donovan Walker

**Affiliations:** 1 Columbia University Irving Medical Center Department of Medicine Division of Endocrinology NY New York USA Division of Endocrinology, Department of Medicine, Columbia University Irving Medical Center, New York, NY, USA

**Keywords:** Microarchitecture, gluten, fracture, bone density, inflammation

## Abstract

Celiac disease (CD) is an autoimmune disorder characterized by small intestinal inflammation triggered by gluten ingestion in genetically-predisposed individuals. A frequent extra-intestinal manifestation of CD is metabolic bone disease which contributes to an increased risk of fracture. The mechanisms underlying bone disease in CD remain incompletely understood, but multiple processes have been proposed including (1) malabsorption of calcium and vitamin D leading to secondary hyperparathyroidism and increased skeletal resorption, (2) pro-inflammatory cytokines altering the osteoprotegerin and receptor activator of nuclear kappa-B ligand ratio favoring osteoclastogenesis, (3) hypogonadism, and (4) low weight and malnutrition. Most studies show reduced bone mineral density in patients with CD. Bone microarchitecture is also deteriorated leading to reduced whole bone stiffness. Many, but not all investigations, have shown an increased risk of fracture associated with CD. The main stay of therapy for CD is maintaining a gluten-free diet. Improvement in bone mineral density with adherence to a gluten-free diet has been well-established. Bone mineral density remains lower, however, compared to controls and increased fracture risk can persist. There is no consensus on the timing of dual-energy x-ray absorptiometry for bone mineral density assessment in patients with CD. Routine screening for CD in patients with osteoporosis is not recommended. Little data are available on the use or efficacy of prescription osteoporosis therapeutics in patients with CD. Studies are needed to develop standardized guidelines for screening and treatment of metabolic bone disease in patients with CD to identify those who may need early intervention with prescription osteoporosis therapy. Arch Endocrinol Metab. 2022;66(5):756-64

## INTRODUCTION

Celiac disease (CD) is a systemic autoimmune disorder characterized by small intestinal inflammation triggered by gluten ingestion in genetically predisposed individuals. Gluten is a protein complex found in various grains including wheat, rye, and barley (1). Ingestion of gluten and its immunogenic fragment, gliadin, leads to an inflammatory cascade that may result in changes in gut permeability, production of pro-inflammatory cytokines, lymphocytic infiltration of the intestinal epithelium and ultimately villous atrophy (2,3). Similar to other autoimmune disorders, CD has a strong genetic component evidenced by familial clustering and high concordance in identical twins (4,5). Human leukocyte antigen (HLA) DQ2 and DQ8 haplotypes play a pivotal role in CD with 99% of CD patients carrying these alleles. HLA-DQ2/HLA-DQ8 is present in up to 30%-40% of the general population, however, only a minority of these patients have CD (1%-3%), suggesting additional factors contribute to the development of disease. Genome wide association studies have identified several non-HLA genes associated with CD (2,6). Environmental factors may be important as well.

CD is estimated to affect 1%-2% of the population worldwide with increasing incidence and prevalence over the last few decades (7–9). CD is more common in women than men (female:male ratio of 2:1 to 3:1). First- and second-degree relatives of patients with CD are at increased risk for developing the disease, with the highest risk in siblings, followed by offspring and parents (10). Patients can present at any age, but most typically present in the first two years of life or in the second to third decade. Classic intestinal symptoms of malabsorption including severe weight loss, chronic diarrhea, and failure to thrive are infrequent and are primarily seen in children. Adults can present with mild abdominal pain or bloating, but may be asymptomatic or have only extra-intestinal symptoms (11,12). The broad spectrum of CD disease and symptoms has been classified using the Oslo definitions of CD and CD-related terms (Table 1).

**Table 1:** Oslo Definitions for Celiac Disease and Celiac Disease-related terms (8)

Classical	Signs and symptoms of malabsorption including failure to thrive, weight loss and diarrhea with or without steatorrhea
Non-classical	No signs or symptoms of malabsorption. Extra-intestinal manifestations including iron deficiency anemia, constipation, bloating, neurologic symptoms, abnormal liver biochemistry, infertility, delayed puberty, fatigue
Subclinical	CD below clinical detection threshold, no signs or symptoms sufficient to prompt routine CD testing
Symptomatic	Clinically evident gastrointestinal or extra-intestinal symptoms in patient with gluten intake
Asymptomatic	No gastrointestinal or extra-intestinal symptoms at time of diagnosis
Potential	Positive CD serology with normal small intestinal mucosa
Refractory	Recurrent or persistent symptoms and villous atrophy after 12 months of strict gluten free diet

The preferred screening test for CD in adults following a gluten-containing diet is measurement of Immunoglobulin A (IgA)-tissue transglutaminase antibodies (tTG-IgA) with a concurrent IgA level. Serologic testing involves measurement of autoantibodies to tTG-IgA or anti-endomysial antibody (EMA-IgA) which target tissue transglutaminase, the autoantigen in CD. Deamidation of gliadin by the enzyme tTG enhances its immunogenicity via increased binding to HLA-DQ2/DQ8. EMA-IgA is highly specific for CD; however, it is not the first-line test due to high cost and operator dependency. Previously used “first generation” anti-gliadin antibody assays are no longer recommended due to their lower diagnostic accuracy. Patients with low IgA levels should have Immunoglobulin G (IgG)-based testing which includes tTG-IgG and deamiated gliadin peptide (DGP)-IgG, as IgA deficiency may lead to falsely negative tTG-IgA (13,14). All diagnostic testing should be done while patients are following a gluten-containing diet. The diagnosis can be established definitively in adults with positive serology by endoscopic evaluation and biopsy. Histologic changes are graded along a spectrum using the Marsh-Oberhuber classification. Duodenal biopsy with increased intraepithelial lymphocytes (Marsh I), crypt hyperplasia (Marsh II) and/or villous atrophy (Marsh III) in a patient with positive serology confirms the diagnosis of CD (15). HLA testing may be helpful in certain circumstances such as in those with discordant serology and histology, those who refuse endoscopy, those following a GFD with negative serologies or those at high risk.

The mainstay of treatment is lifelong adherence to a gluten-free-diet (GFD), which enables villous healing. There is currently no food and drug administration-approved medical therapy for CD, but several treatments are under investigation. These include several drugs in Phase II trials that address the inflammatory response, inhibit the TTG enzyme, as well as a vaccination aimed at desensitizing to gluten. Immune modulators include PRV-015, a monoclonal antibody targeting IL-15 which is thought to be a key factor in CD pathology, as well as TAK-101 (16), gliadin-encapsulated nanoparticles that induce immune tolerance to gluten. ZED1227 is a transglutaminase inhibitor and latiglutenase, is an enzyme which breaks down gluten, making it nontoxic in CD patients and can be supplemented with CD diet. Larazotide, a tight junction regulator which decreases intestinal permeability, was being studied in a Phase III trial that has been discontinued (17).

One of the most common extra-intestinal manifestations of CD is metabolic bone disease (MBD) which contributes to an increased risk of fracture in patients with CD (18–20). Many studies indicate that patients with CD, including those with asymptomatic disease (21), have lower bone mineral density (BMD) compared to age- and sex-matched controls (22,23). The reported prevalence of osteopenia or osteoporosis in CD is variable, ranging from 38%-72% of newly diagnosed patients (Table 2) (24–27). In a large cohort of patients with CD age 50 years and older, 44% had osteoporosis, indicating that this is a frequent and important issue for this population (28). This review will provide an overview on MBD related to CD in adults.

**Table 2:** Prevalence of osteopenia and osteoporosis in recent adult celiac disease cohorts

Author and Year of Publication	Study Design	Participants with CD	Osteoporosis^+^ (%)	Osteopenia (%)
Valdimarsson (39), 1996*	Prospective	63	LS: 15% Hip: 18% FA: 22%	
Meyer (42), 2001	Cross-sectional	128 (105/23)	LS: 38% FN: 27% FA: 36%	LS: 38% FN: 44% RA: 32%
Galli (41), 2018	Cross-sectional	214 (153/61) Age: median (range) 38 (18.72)	LS and/or FN: 17.8%	LS and/or FN: 42.5%
Ganji (24), 2019	Meta-analysis	563	LS: 16.3% Hip: 13.3% LS and FN: 14.4%	LS: 41.9% Hip: 46.9% LS and FN: 39.6%
Walker (28), 2020	Cross-sectional	721 (493/228) Age: mean (range) 43.6 (17-83)	At spine, hip or forearm: 19.6%	At spine, hip or forearm: 43.3%
Sayar (25), 2021	Cross-sectional	100 (84/16) Age: Mean 34.6 years	LS: 15.2% FN: 10.8%	LS: 67.3% FN: 43.4%

LS: lumbar spine; FN: femoral neck; RA: radius. *Authors use T-score < -2.0. + Based on T-score < -2.5

### Pathophysiology of metabolic bone disease in celiac disease

**Figure 1:**
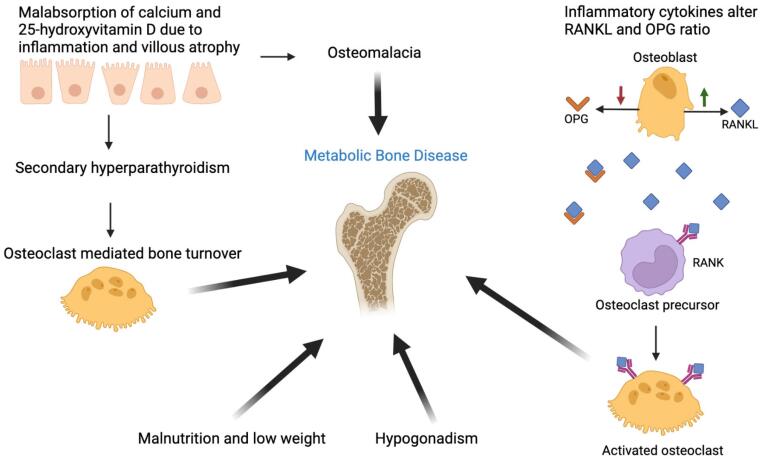
Schematic of potential pathophysiological mechanisms contributing to metabolic bone disease in celiac disease.

The pathophysiological mechanisms underlying MBD in CD remain incompletely understood, but multiple processes have been proposed (Figure 1). Early in CD, villous atrophy and/or inflammation in the small intestine, the principal site for calcium and vitamin D absorption, is hypothesized to lead to malabsorption of these nutrients. In some patients, hypocalcemia and/or vitamin D deficiency may lead to secondary hyperparathyroidism and subsequent osteoclast-mediated bone turnover (29). Limited data suggest, up to 25% of patients develop secondary hyperparathyroidism and increased bone resorption (30,31). Despite a GFD, absorption of calcium may remain reduced. In one study, even four years after institution of a GFD, fractional absorption of calcium remained lower in patients with versus without CD (32). Osteomalacia, or impaired bone mineralization, is possible if malabsorption is prolonged. Limited information exists regarding the prevalence, but osteomalacia appears to be relatively rare among adults in the United State today. In one study of 103 patients, 21% had 25-hydroxyvitamin d levels less than 20 ng/mL and alkaline phosphatase (a possible indicator of osteomalacia) was elevated in about 10% of patients (33).

Inflammation is also thought to contribute to bone loss in CD (29). Pro-inflammatory cytokines may contribute to osteoclastogenesis via an imbalance in the receptor activator of nuclear kappa-B ligand (RANKL) pathway (34,35). Binding of RANKL to RANK on osteoclast precursors causes differentiation to mature osteoclasts and increases bone resorption. The ratio of osteoprotegerin (OPG), a decoy receptor blocking RANKL-RANK binding, and RANKL determines the degree of osteoclastogenesis. Inflammatory cytokines lower the ratio favoring osteoclastogenesis (36,37). Limited data suggest altered OPG/RANKL ratios in CD, with lower ratios associated with lower spinal BMD (38). Other potential contributors include hypogonadism, low weight and malnutrition. Low insulin-like growth factor-1 (IGF-1) may also play a role (28).

### Effect of celiac disease on bone mineral density

Several studies evaluating BMD have shown reduced BMD in patients with CD, but there is significant heterogeneity in results due to varying methods of data collection, analysis of different skeletal sites, differences in age ranges included and duration of disease or GFD. A prospective study in 63 men and women found low forearm, trochanter, and spine BMD in 22%, 18% and 15% of patients, respectively (39). Pistorius and cols. found only reduced femoral neck (FN) BMD in pre-menopausal women with CD but low FN and spine BMD in post-menopausal women with CD (40). Galli and cols. found 60% of patients with CD had reduced BMD: 42.5% with osteopenia and 17.8% with osteoporosis. Age greater than 45 years, male gender and low weight was associated with osteoporosis (41). Other studies also found low BMD, particularly in men. Meyer and cols. reported osteoporosis in 34% of patients at the spine, 44% at the FN, 32% at the radius and found men were more severely affected than women when compared to age-matched controls (42). A large study found 5% of patients with CD had osteoporosis limited to the one-third radius (28). In men, the one-third radius was the most frequent site for osteoporosis, highlighting the importance of evaluating this skeletal site in this population. In this study, males were more likely to have osteoporosis, forearm osteoporosis and lower Z-scores at the spine and forearm compared to women (28). Further, greater degree of villous atrophy was associated with lower T-score and Z-score at the one-third radius. Patients with total versus partial atrophy were more likely to have osteoporosis at any site and were more likely to be male. This suggests lower BMD in men versus women may relate to greater severity of disease possibly related to delays in diagnosis, but this requires further study.

### Skeletal microstructure in celiac disease

**Figure 2:**
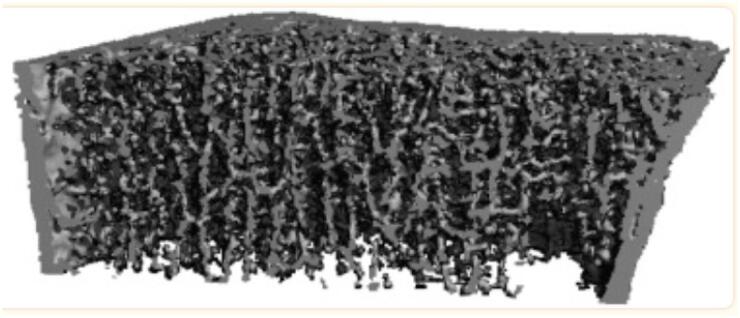
Representative HR-pQCT scan of a study participant with celiac disease illustrating trabecular defects and cortical thinning, adapted from Stein and cols. (44).

Few studies have investigated bone microarchitecture in CD. High resolution peripheral quantitative computed tomography (HRpQCT) non-invasively measures volumetric bone density and skeletal microstructure, providing separate measures for trabecular and cortical bone. A study in 31 pre-menopausal women showed significant deterioration in trabecular and cortical indices in patients with CD versus controls. Radial trabecular density was 26% lower and trabeculae were thinner, fewer and more widely spaced. Cortical density was reduced by 4%. Similar findings were observed at the distal tibia. The microarchitectural deficits were greater in those with symptomatic compared to subclinical CD (43). A subsequent study confirmed these findings and also found reduced whole bone stiffness (a biomechanical indicator of strength, which is associated with fracture) measured by microfinite elemental analysis (FEA) compared to controls (44) (Figure 2). There is no comparable HRpQCT data in men with CD. Based on studies using DXA and HRpQCT, both the quantity and quality of bone appear to be affected by CD.

### Fracture risk in celiac disease

As suggested by studies indicating reduced BMD, many, but not all investigations, have shown an increased risk of fracture associated with CD (Table 3). Vasquez and cols. found a high prevalence of peripheral skeletal fractures in patients with CD versus controls (25% vs. 8%) with a majority of fractures occurring at the wrist and forearm (20), while Ludvigsson and cols. found increased risk of hip fracture for up to 20 years after CD diagnosis (45). In contrast, studies from Denmark (46) and the UK (47) were not able to document an increased risk of fracture associated with CD and there was no difference in fracture risk before or after CD diagnosis. On the other hand, another study showed patients with CD had an increased relative risk of any fracture (30% increase), hip fracture (90% increase), and ulna or radius fracture (77% increase) but the increase in absolute risk was small (48). Symptomatic men and women with CD had significantly higher rates of fractures and risk of first peripheral fractures compared to controls and those with non-classical and subclinical CD in two studies (49,50). Importantly, patients presenting with gastrointestinal symptoms were found to have a higher risk of fracture than those without symptoms. Males with CD had a greater total number of fractures and younger age at the time of first fracture compared to women with CD (50). Meta-analyses support findings of increased fracture risk in patients with CD. One such analysis that included eight studies with over 20,000 patients found a 43% greater risk of fracture in patients with CD (51). Similarly a second meta-analysis indicated risk for any fracture and hip fracture were increased by 30% and 69% respectively in those with CD (52). Thus, the majority of data indicate fracture risk is increased in CD, and symptomatic patients as well as men may be most at risk.

**Table 3:** Selected studies of fracture risk in celiac disease

Author and Year of Publication	Study Design	Participants – CD (Female/Male)	Participants – Controls (Female/Male)	Fracture Outcomes	Adjusted OR/HR (95% CI)
Vasquez (20), 2000	Case-control	165 (143/22) Age: Mean (range) years 40 (16-74)	165 (143/22) Age: Mean (range) year 41 (16-74)	All Spine	3.5 (1.8-7.2) 2.8 (0.7-11.5)
Vestergaard and Mosekilde (46), 2002	Prospective case-control	1021 CD (588/433) Age: Mean 31 years	3063 (1764/1299) Age: Mean 31 years	Any fracture before or after diagnosis	0.70 (0.45-1.09) 0.94 (0.7-1.24)
Thomason (47), 2003	Case-control	244 (171/73) Age: Mean (range) years 60 (<55-75+)	161 (115/46) Age: Mean (range) years 61 (<55-75+)	Low trauma All Hip Forearm	1.16 (0.65-2.10) 1.05 (0.68-1.62) 0.66 (0.05-9.50) 1.21 (0.66-2.25)
West (48), 2003	Case-control	4732 (3095/1637) Age: mean 43.5 years	23,620 (18,545/5075) Age: mean 43.5 years	All Hip Radius	**1.3 (1.16-1.46)** **1.9 (1.20-3.02)** **1.77 (1.35-2.34)**
Moreno (49), 2004	Case-control	Classical: 78 (62/16) Age: mean (range) 44 (18-77) NC/SC: 70 (55/15) Age: mean (range) 38 (17-81)	296 (236/60) Age: mean 41 years	All Classical CD NC/SC CD	3.6 (1.7-7.5) 5.2 (2.8-9.8) 1.7 (0.7-4.4)
Ludvigsson (45), 2007	Retrospective Cohort (adults and children)	14,187 (8311/5876) Age: Median (range) 53 (0-93)	68,952 (40,430/28,522) Age: Median (range) 53 (0-93)	All Hip	**1.4 (1.3-3.5)** **2.1 (1.8-2.4)**
Olmos (51), 2008	Meta-analysis	23,955	96,777	All	**1.43 (1.15-1.78)**
Sanchez (50), 2011	Case-control	265 (223/42) Age: Median (range) 42 (18-85)	530 (446/84) Age: median (range) 43 (16-87)	Peripheral before CD diagnosis Peripheral after CD diagnosis	1.78 (1.23-2.56) 1.28 (0.74-2.21)
Heikkila (52), 2015	Meta-analysis			All Hip	1.92 (1.29-2.84) 1.75 (0.78-3.89)

NC: non-classical; SC: subclinical. Bold indicates statistically significant associations.

### Bone mineral density response to treatment with a gluten-free diet

Improvement in BMD after strict adherence to a GFD has been well-established, however, BMD remains lower compared to disease-free controls and increased fracture risk can persist (39,53,54). Some studies indicate that BMD increases by 5%-8% on average with most improvement in BMD seen within the first one to three years of GFD initiation (55,56). There is, however, wide inter-individual variability. Predictors of BMD improvement remain to be elucidated. One study found that patients with lower baseline serum calcium had greater improvement in BMD suggesting those with the greatest calcium malabsorption had the greatest gains in BMD (33). Others suggest symptoms (56), age (57) and menopause status (53), may affect the response to a GFD. A few studies showed decreased fracture risk with adherence to a GFD (20,50), however, several other studies did not find a significant difference in fractures before and after GFD initiation (48). In a prospective study of >7,000 patients with biopsy-proven histologic evidence of CD who underwent repeat endoscopy, persistent villous atrophy (versus mucosal healing) was associated with an increased risk of hip fracture. These results indicate adherence to a GFD that allows for mucosal healing may reduce the risk of fracture. Given this data, patients should be counseled about the importance of GFD with regard to skeletal health (58).

Similarly, skeletal microstructure improves with adherence to a GFD. Using HRpQCT, improvement in both trabecular and cortical parameters were seen after one year of a GFD. The trabecular compartment had a greater increase compared to the cortical compartment (9% vs. 0.4% at the distal radius and 8% vs 1.5% at the distal tibia), primarily driven by trabecular thickness. This was hypothesized to be due to the higher remodeling rate in the trabecular compartment. Despite improvement, a majority of the bone parameters remained significantly lower compared to the healthy control group (59). Improvement in trabecular and cortical parameters was, however, maintained 3 years later (60).

### BMD assessment in patients with celiac disease

There is no consensus on the timing of DXA for the assessment of BMD in patients with CD and guidelines vary by society. The International Society for Clinical Densitometry recommends BMD testing at diagnosis only in adults with classic malabsorptive symptoms (61). In contrast, the European Society for Study of Celiac Disease recommends BMD testing at time of diagnosis in all adult CD patients (62). The American Society of Gastroenterology does not recommend for or against BMD testing. A recent Canadian position statement suggests BMD assessment at diagnosis in patients with classic CD; however, in those with asymptomatic or subclinical CD, they suggest BMD be evaluated one year post-initiation of a GFD (63). Similarly, the American Gastroenterological Association (AGA) recommends BMD assessment one year after GFD initiation in newly diagnosed CD patients (64), while the British Society of Gastroenterology (BSG) recommends BMD testing after one year of a GFD in patients above age 55 or those who have additional risk factors for osteoporosis (65). In patients with additional risk factors including post-menopausal status, age over 50 and history of fragility fracture, earlier screening has been suggested by some authorities. If osteoporosis or osteopenia is detected on an initial screening DXA scan, a follow up DXA scan is recommended in one year (63). In patients with normal baseline BMD, testing can be repeated in two to three years (63).

In some regions, implementation of these guidelines is suboptimal and DXA is under-utilized. For example, a retrospective study in the United States found only 36% of patients with CD in an outpatient tertiary referral center were appropriately referred for DXA as per AGA guidelines (66). On the other hand, in Australia, a recent study found that 82% of patients with CD were screened for low BMD (67). All patients with CD should be evaluated for vitamin D deficiency by measurement of serum 25-hydroxyvitamin D (63,68). Measurement of PTH can be considered based on clinical factors (such as severity of vitamin d deficiency, osteoporosis, presence of osteomalacia or hypocalcemia, etc.).

### Testing for celiac disease in patients with osteoporosis

Routine screening for CD is not recommended in all adults with osteoporosis. The optimal approach is, however, unclear due to conflicting data. Stenson and cols. identified a higher prevalence of CD among post-menopausal women with osteoporosis (3.4%) compared to those without osteoporosis (0.2%) and recommended routine serologic screening for CD in all patients with osteoporosis (69). More recent studies, including a meta-analysis in over 3000 patients found only 1.6% of patients with osteoporosis have biopsy-proven CD (70), a rate comparable to that of the general population. This implies routine screening for CD in those with osteoporosis may be low yield, especially in patients without gastrointestinal symptoms (71,72). A Canadian multidisciplinary task force recommends an individualized approach especially in patients with gastrointestinal symptoms, those with a family history of CD, or when vitamin D insufficiency, low urinary calcium level or unexplained anemia are present (73). In contrast, there are some subgroups in whom the suspicion for CD is high and testing should be undertaken in such individuals. Pre-menopausal women and men below age 70 with osteoporosis, those with Z-scores two or more SD below age-matched controls, those with worsening osteoporosis despite therapy or without risk factors should be evaluated for secondary causes of osteoporosis, including CD (74).

### Treatment of osteoporosis in patients with celiac disease

CD-associated bone demineralization and osteomalacia, if present, must be treated with initiation of a GFD and appropriate supplementation with calcium and vitamin D. However, as discussed above, initiation and maintenance of a GFD improves, but may not normalize, BMD. In post-menopausal women and older men with osteoporosis or fragility fractures, prescription therapy for osteoporosis should be considered while maintaining a GFD to reduce the risk of fracture. The optimal timing of prescription therapy after initiation of a GFD is unclear. Given BMD tends to increase in the first year following initiation of a GFD, it may be reasonable to delay therapy in many low risk patients with osteoporosis (particularly those who are young or without fracture) until 1 year after a GFD has been started and BMD has been reassessed. Clinicians should ensure 25-hydroxyvitmamin D levels are in the sufficient range, that osteomalacia and hypocalcemia are not present and calcium intake and absorption are adequate prior to initiation of medications for the treatment of osteoporosis (75). The AGA recommends initiating bisphosphonates in adults with celiac disease in the setting of osteoporosis, vertebral compression fractures or osteopenia with prolonged corticosteroid use.

Little data are available on the use or efficacy of prescription osteoporosis therapy in patients with CD. Parenteral, rather than oral, therapies may be preferable if absorption is a concern or gastrointestinal symptoms persist. Stuckey and cols. observed that 44% of patients with CD and low BMD or osteoporosis were prescribed osteoporosis therapy including alendronate, risedronate, zoledronic acid, raloxifene, strontium, denosumab and teriparatide. The most commonly utilized therapy was bisphosphonates (67). A randomized controlled trial in 28 osteopenic and osteoporotic patients with CD did not find a significant improvement in BMD with combination zoledronic acid and a GFD compared to a GFD alone, but was undoubtedly underpowered to detect such a difference (76). The most commonly reported side effects with oral bisphosphonates are gastrointestinal symptoms. Case reports, however, have described rare episodes of hypocalcemia in patients with CD being treated with bisphosphonates. Risk factors for hypocalcemia, including vitamin D deficiency, increased bone resorption, hyperparathyroidism and hypomagnesemia, are also commonly seen in untreated CD patients (77,78).

### Summary and future directions

In conclusion, low BMD and osteoporosis are common in patients with CD and suggest that fracture risk is increased. The optimal approach to screening for MBD in patients with CD is unclear and recommendations from different societies are inconsistent. Not all societal guidelines endorse BMD testing in all adults with CD and testing is inconsistent in practice; as such, some at risk may fail to be identified. The foundation of addressing bone disease related to CD is initiation and adherence to a GFD and ensuring adequate calcium and vitamin D intake. Currently, very few studies have assessed which patients have the greatest increase in BMD after initiation of a GFD. Such data may assist with identification of those at risk for persistent bone disease if treated with a GFD alone. Lastly, men with CD may be particularly susceptible to bone loss and fracture, but limited data exist. Future studies are needed to develop standardized guidelines for the screening and treatment of bone disease in patients with CD in order to identify high risk patients who may need early intervention with osteoporosis therapy.
